# Comprehensive Analysis of the *COBRA-Like* (*COBL*) Gene Family in *Gossypium* Identifies Two *COBL*s Potentially Associated with Fiber Quality

**DOI:** 10.1371/journal.pone.0145725

**Published:** 2015-12-28

**Authors:** Erli Niu, Xiaoguang Shang, Chaoze Cheng, Jianghao Bao, Yanda Zeng, Caiping Cai, Xiongming Du, Wangzhen Guo

**Affiliations:** 1 State Key Laboratory of Crop Genetics & Germplasm Enhancement, Hybrid Cotton R & D Engineering Research Center, Ministry of Education, Nanjing Agricultural University, Nanjing, Jiangsu, China; 2 State Key Laboratory of Cotton Biology, Cotton Research Institute, Chinese Academy of Agricultural Sciences, Anyang, Henan, China; Zhejiang A & F university, CHINA

## Abstract

*COBRA*-*Like* (*COBL*) genes, which encode a plant-specific glycosylphosphatidylinositol (GPI) anchored protein, have been proven to be key regulators in the orientation of cell expansion and cellulose crystallinity status. Genome-wide analysis has been performed in *A*. *thaliana*, *O*. *sativa*, *Z*. *mays* and *S*. *lycopersicum*, but little in *Gossypium*. Here we identified 19, 18 and 33 candidate *COBL* genes from three sequenced cotton species, diploid cotton *G*. *raimondii*, *G*. *arboreum* and tetraploid cotton *G*. *hirsutum* acc. TM-1, respectively. These *COBL* members were anchored onto 10 chromosomes in *G*. *raimondii* and could be divided into two subgroups. Expression patterns of *COBL* genes showed highly developmental and spatial regulation in *G*. *hirsutum* acc. TM-1. Of them, *GhCOBL9* and *GhCOBL13* were preferentially expressed at the secondary cell wall stage of fiber development and had significantly co-upregulated expression with cellulose synthase genes *GhCESA4*, *GhCESA7* and *GhCESA8*. Besides, *GhCOBL9* D_t_ and *GhCOBL13* D_t_ were co-localized with previously reported cotton fiber quality quantitative trait loci (QTLs) and the favorable allele types of *GhCOBL9* D_t_ had significantly positive correlations with fiber quality traits, indicating that these two genes might play an important role in fiber development.

## Introduction

Cellulose, composed of long parallel linear β-1, 4-D-glucan chains, is the major component of the primary and secondary cell walls of plants. Studies on cellulose biosynthesis will not only facilitate an understanding of cell wall development but also open the possibility to increase the economic value of cotton fiber. Over the past decades, several genes essential for cellulose biosynthesis have been revealed, such as *cellulose synthase* (*CESAs*), *KORRIGAN* (*KOR*), *chitinase-like* genes (*CTLs*), *fasciclin-like arabinogalactan* genes (*FLA*s), *NAC* and *MYB* transcription factors [1–9]. Although these important findings have been made, the molecular mechanism for cellulose biosynthesis and deposition remains largely unknown.

Besides the importance of cotton fiber to the textile industry, it is also considered to be an ideal model to investigate the mechanism of cell elongation and cellulose deposition [10–11]. The fiber cell origins from the epidermis of ovules and is a highly elongated and thickened single-cell trichome with >90% crystalline cellulose in mature fibers. Fiber formation involves four distinct but overlapping stages [12–13]: initiation (-3 days post anthesis [DPA] to 3 DPA), elongation (3 DPA to 23 DPA), secondary cell wall synthesis (16 DPA to 40 DPA) and maturation (40 DPA to 50 DPA), which collectively determine the fiber yield and quality traits. Disorders of any stages will affect the quality traits of fiber such as fiber length, strength, micronaire, elongation and fiber uniformity [14–15]. As cellulose accounts for about 35% in primary cell wall and more than 90% in secondary cell wall of cotton fiber, it plays an important role in fiber elongation and secondary cell wall formation [16]. Especially during the secondary cell wall synthesis stage, almost pure cellulose is produced in cotton fiber cell. Thus cotton fiber provides an excellent model for mining and characterization of key genes related to biosynthesis and assembly of cellulose. On the other side, studies on cellulose biosynthesis in cotton fiber will also contribute to improving fiber quality and yield.

*COBRA*-*Like* genes (*COBL*s), which encode the glycosylphosphatidylinositol (GPI) anchored proteins, have been reported to play significant roles in the orientation of microfibrils and cellulose crystallinity status. The *COBL* family has been identified in *Arabidopsis thaliana* [17], *Oryza sativa* (*BC1*-*Like*) [18], *Zea mays* (*BK2*-*Like*) [19] and *Solanum lycopersicum* [20]. As a result, 12, 11, 9 and 17 members were found in these plants respectively. The *COBL* members are characterized by an N-terminal signal peptide, a carbohydrate-binding module (CBM), a highly conserved central cysteine-rich domain (CCVS) and a C-terminal domain including a GPI modification motif and a hydrophobic tail for targeting the protein to the endoplasmic reticulum. The *COBL* family is divided into two groups with the first group similar to *COBRA* and the second group to *COBL7* in *A*. *thaliana* [17]. The mutant *cobra* in *A*. *thaliana* causes the defects in polar longitudinal expansion in root cells [21–23] and the mutant *bc1l4* in *O*. *sativa* also shows a typical dwarf phenotype [24]. Similarly male gametophyte transmission in *bc1l5* mutants is severely altered and specifically blocked [18] and the root hair of *rth3* mutants in *Z*. *mays* initiates primordia but fails to elongate properly [25]. *COBL* genes are largely responsible for the secondary cell wall biosynthesis. The mutations *cobl4* in *A*. *thaliana*, *brittle culm 1* (*bc1*) in *O*. *sativa* and *brittle stalk 2* (*bk2*) in *Z*. *mays* affect the mechanical strength of vascular bundles and have a significant reduction in cellulose content [26–28]. In addition, Liu et al. [29] demonstrated that *COBL* proteins could modulate cellulose structure by binding to cellulose and further affecting microfibril crystallinity. Whereas, it still needs to reveal the underlying molecular basis that how the *COBLs* proteins have functions in cellulose biosynthesis.

Recently the publically genomic information from three sequenced cotton species including the closest extant progenitor relatives for tetraploid cotton species, D-genome *Gossypium raimondii* [30], A-genome *Gossypium arboreum* [31], upland cotton genetic standard line *Gossypium hirsutum* acc. TM-1 [32], generates a solid foundation for characterizing gene families at a genome-wide level. Here we performed the first comprehensive investigation of the *COBL* gene family in three sequenced cotton species involving in the analysis of sequence phylogeny, genomic structure, chromosomal location and adaptive evolution. Further the expression patterns of *COBL* genes in *G*. *hirsutum* acc. TM-1 in various tissues/organs and different developmental stages of fiber development were elucidated by integrating RNA-Seq data and quantitative real-time PCR (qRT-PCR) analysis. Based on the co-expression analysis, correlation analysis and integration of quantitative trait loci (QTLs), we verified that two *COBLs*, *GhCOBL9* and *GhCOBL13* were significantly associated with fiber quality. This study will open up the possibility of exploring the use of *COBLs* to improve fiber quality in future cotton-breeding programs.

## Materials and Methods

### Database search and identification of *COBL* genes in *Gossypium*

The genomic database of three cotton species *G*. *raimondii*, *G*. *arboreum* and *G*. *hirsutum* acc. TM-1 were downloaded from http://www.phytozome.net/, http://cgp.genomics.org.cn and http://mascotton.njau.edu.cn/ respectively. The protein database of *A*. *thaliana*, *O*. *sativa* and *Z*. *mays* were obtained from The Arabidopsis Information Resource (TAIR: http://www.arabidopsis.org), the Rice Genome Annotation Project Database (RGAP release 7, http://rice.plantbiology.msu.edu/index.shtml) and http://www.phytozome.net/, respectively.

*COBL* domain (PF04833) was downloaded from Pfam (http://pfam.xfam.org/) and the genes shared with the domain could be obtained by taking PF04833 as the query to scan individually the three cotton protein database using the HMMER software version 3.0 [33] with the default parameters (E value < 0.01). Further, to confirm the genes above, gene prediction programs FGENESH (http://www.softberry.com/berry.phtml) and SMART (http://smart.embl-heidelberg.de/) were performed to detect the conserved motifs. Finally, the paralogs of *COBLs* in the three cotton species were confirmed by BLASTp.

### Chromosomal locations and gene duplications

Chromosomal location of *COBL* genes in *G*. *raimondii* was performed using MapInspect software (http://www.plantbreeding.wur.nl/UK/ software_mapinspect.html). The nomenclature and description of the distribution of genes in chromosomes were derived from the map constructed by Zhao et al. [34]. In other two cotton species *G*. *arboreum* and *G*. *hirsutum* acc. TM-1, the nomenclature of *COBL* genes was following its ortholog in *G*. *raimondii*. DNAMAN (http://www.lynnon.com/) was conducted to calculate the sequence similarity among the *COBL* genes. Tandem duplicates were defined as genes separated by five or fewer genes and segmental duplicates were identified using the Plant Genome Duplication Database (http://chibba.agtec.uga.edu/duplication) [35]. The ratios of nonsynonymous to synonymous substitutions (Ka/Ks) were then analyzed to assess the selection pressure for the identified paralogous pairs [36].

### Gene structure and phylogenetic analysis

MEGA 5.0 software (www.megasoftware.net) was used to construct the phylogenetic tree following the maximum likelihood method [37]. The parameters used were as follows: Test of phylogeny: Bootstrap method, No. of bootstrap replications: 1000, substitutions type: Amino acid, model/method: Jones-Taylor-Thornton (JTT) model, rates among sites: Uniform rates, gaps/missing data treatment: Complete deletion, ML heuristic method: Nearest-Neighbor-Interchange (NNI), initial tree for ML: Make initial tree automatically. The online ExPASy tool (http://www.expasy.org/tools/) and Gene Structure Display Server 2.0 (http://gsds.cbi.pku.edu.cn) were used to identify the physicochemical parameters [38] and the exon/intron organization. The alignment of the *COBL* family members was carried out with ClustalX 1.83 software [39].

Conserved motif annotation was performed using the MEME program [40]. If two or more motifs identified with SMART program represented the same domain and were located adjacently, they would be merged into one domain district. The parameters of the MEME program were as follows: Number of repetitions: Any, maximum number of motifs: 10, the optimum motif widths: between 4 and 50 residues. Furthermore the signal peptide, hydrophobic plot and GPI modification motif (ω site for dissociation) were predicted by the SignalP 4.1 Server (http://genome.cbs.dtu.dk/services/SignalP/), KYTE DOOLITTLE HYDROPATHY PLOT (http://gcat.davidson.edu/DGPB/kd/kyte-doolittle.htm) and the big-PI predictor (http://mendel.imp.ac.at/sat/gpi/gpi_server.html) [41] respectively.

### Plant materials and DNA/RNA isolation

The field evaluations of TM-1 and Hai7124 were conducted in PaiLou experimental field, Nanjing Agricultural University, Jiangsu Province, China. All necessary permits for collecting TM-1 and Hai7124 were obtained from Nanjing Agricultural University, China. The field evaluations of natural population including 285 *G*. *hirsutum* and 4 *G*. *barbadense* cultivars or lines were conducted in the experiment field of three Ecological Stations of the Institute of Cotton Research, CAAS. All necessary permits for the field evaluations of these accessions were obtained from the Institute of Cotton Research, CAAS, China. All the field evaluations were not relevant to human subject or animal research. Therefore, they did not involve any endangered or protected species.

*G*. *hirsutum* acc. TM-1, the genetic standard line of upland cotton and *G*. *barbadense* cv. Hai7124 with superior fiber traits were used for gene cloning. *G*. *hirsutum* acc. TM-1 were also employed to carry out the expression analysis. Among them, vegetative tissues (roots, stems and leaves) were collected from two-week-old seedlings; Floral tissues (petals and anthers) were collected on the day of flowering; Fibers were sampled on the different days post anthesis. All samples were quick-frozen in liquid nitrogen and stored at -70°C before use.

Natural population including 285 *G*. *hirsutum* and 4 *G*. *barbadense* cultivars or lines (S1 Table) were collected mainly from China and some from foreign countries, which were available from the National Mid-term Genebank of the Institute of Cotton Research, Chinese Academy of Agricultural Sciences (CAAS). These accessions were grown in three Ecological Stations of the Institute of Cotton Research with three replicates: Kuche of Xinjiang province (northwestward cotton growing region), Anyang of Henan province (Yellow River valley cotton growing region) and Nanjing of Jiangsu province (Yangtze River valley cotton growing region) during 2007 to 2009. After harvesting of the plants, the fiber samples including three biological replicates were tested with HVI spectrum in the Supervision, Inspection and Test Center of Cotton Quality, Ministry of Agriculture in China. The analysis of fiber quality traits focused mainly on 2.5% fiber span length (FL), fiber strength (FS), fiber micronaire (FM), fiber elongation (FE) and fiber uniformity (FU).

Total DNA was extracted from the leaves of seedlings as described by Paterson et al. [42]. All samples were quantified using “one drop spectrophotometer OD-1000+” (OneDrop, Nanjing, China) and adjusted to a concentration of 20–60 ng/μL. Total RNA was isolated from all samples with CTAB [43] and the trizol of “TransScript One-Step gDNA Removal and cDNA Synthesis SuperMix” kit (TransGen, Nanjing, China) was used subsequently to obtain the first strand cDNA. The components of each reaction sample included 1 μg RNA, 1 μL of anchored oligo(dT)_18_ primer, 10 μL of 2*TS reaction mix, 1 μL of RT/RI enzyme mix, 1 μL of gDNA remover and additional ddH_2_O to give a total volume of 20 μL. All cDNA samples were stored at -30°C before use.

### Primer design and gene cloning

Primers for PCR amplification of DNA or cDNA for sequencing were designed with Primer Premier 5 (http://www.premierbiosoft.com). Beacon Designer 7.91 software was used to design the primers for qRT-PCR based on the coding sequences of *COBL* members close to the 3’-UTR regions. Simultaneously, the specific primers for “Single Nucleotide Polymorphism (SNP)” of *GhCOBL9* and *GhCOBL13* were designed with the online software WebSNAPER-SNAP (http://pga.mgh.harvard.edu/cgi-bin/snap3/websnaper3.cgi) [44]. All the gene-specific primers were provided in S2 Table.

High-Fidelity ExTaq DNA Polymerase (TaKaRa Biotechnology Co. Ltd. China) was employed to conduct a standard PCR analysis following the manufacturer’s instructions with the amplification programs as follows: Pre-denaturation at 95°C for 5 min, 35 cycles of denaturation at 94°C for 45 s, annealing at 56°C for 45 s and extension at 72°C for 1 min/1 kb with a final extension at 72°C for 10 min. PMD19-T vector (TaKaRa Biotechnology Co. Ltd. China) and *E*. *coli* “strain” Top10 were used for transformation of target fragments. In order to obtain the sequences from both the A-subgenome and D-subgenome, at least 10 clones for each gene from each of the tetraploid species were picked randomly and sequenced with a minimum of three clones was used to determine the gene sequence in each duplicated copy.

### Expression pattern analysis

The high-throughput RNA-sequencing data of *G*. *hirsutum* acc. TM-1 were employed from the accession codes SRA: PRJNA248163 in the National Center for Biotechnology Information (http://www.ncbi.nlm.nih.gov/) including the following tissues: leaves, roots and stems of two-week-old plants; petals and anthers dissected from whole mature flowers; ovules and fiber mixtures from plants -3, -1, 0, 1, 3 DPA and fibers from plants 5, 10, 20 and 25 DPA with three biological replicates. The expression levels of *COBLs* were calculated using the fragments per kilobase of exon model per million mapped reads (FPKM) method by using Cufflinks software with default parameters (http://cufflinks.cbcb.umd.edu/).

qRT-PCR was performed on an ABI 7500 real-time PCR system (ABI Biosystems, http://www.lifetechnologies.com) with the amplification programs as follows: Pre-denaturation at 95°C for 10 min, 40 cycles of denaturation at 95°C for 15 s, annealing at 60°C for 15 s and extension at 72°C for 15 s with a final extension at 72°C for 10 min. Each reaction sample was mixed with 10 μl of SYBR Green Master (Rox), 1.5 μL of cDNA, 5 μM of primers and 7.5 μL of ddH_2_O to give a total volume of 20 μL. For all real-time PCR reactions, three technical replicates were performed in each of the three biological experiments. The relative expression level was calculated using the 2^-△CT^ method [45] and the expression level of the cotton histone3 (AF024716) gene was used as the endogenous control [46].

Pearson correlation analysis on expression pattern was used to calculate the correlation coefficient between RNA-seq and qRT-PCR detection, *GhCESAs* and *GhCOBLs* and different paralog pairs of *GhCOBLs*. For correlation analysis, the total expression of homeologous pairs in each gene from RNA-seq data was merged for matching the result from qRT-PCR detection using gene-specific primer pairs.

### SNP/EcoTilling assays and QTLs integration for function prediction

The amplification program for SNP analysis by PCR was as follows: Pre-denaturation at 95°C for 10 min, 28 cycles of denaturation at 95°C for 15 s, annealing at 62°C for 30 s and extension at 72°C for 30 s followed by denaturing gel electrophoresis. For “EcoTilling” detection, several successive steps were performed following a method modified from that of Zeng et al. [47]. PCR amplification of the natural population (with annealing at 67°C for 30 s) was carried out twice to determine the density and specificity of targeted region with the same forward primer and two different reverse primers. Each sample was then mixed with DNA from the genetic standard line TM-1 in a 1:1 ratio and these were hybridized through 40 cycles of denaturation at 99°C for 10 min and annealing at 72°C with a decrease of 0.3°C per cycle. Finally CELⅠreaction was carried out for 30 min to cleave any mismatches between the query DNA and reference DNA. The denaturing gel electrophoresis after silver staining was used to pinpoint the cleaved fragments with the fragments sizes marked [48]. The cut DNAs were visible as bands and those with faster mobility than the full-length product were considered as polymorphisms. In addition, the DNA fragments around the polymorphic locus were cloned and sequenced to confirm the applicability and accuracy of the polymorphism detection.

Correlation analysis estimated by SPSS18.0 (http://www.spss.com.cn/) was conducted using the LSR (the least significant range) method to study significant difference of fiber quality traits between the two genotypes of targeted genes.

QTLs related to fiber quality traits (including FL, FS, FM, FE and FU) were retrieved and anchored to the *G*. *hirsutum* acc. TM-1 genome. Within ± 5 Mb interval flanking the QTLs, we integrated the QTLs with the prior target genes for association analysis using the MapChart program (www.joinmap.nl.).

## Results

### Genome-wide identification of *COBL* gene family in *Gossypium*

The whole genome sequence scaffolds of three sequenced cotton species (*G*. *raimondii*, *G*. *arboreum* and *G*. *hirsutum* acc. TM-1) were used for the genome-wide exploration of *COBL* family genes in *Gossypium*. Using the *COBL* protein database (PF04833), we searched the three protein database of cotton species by HMMER software. As a result, 19, 18 and 33 *COBL* members were identified respectively in *G*. *raimondii*, *G*. *arboreum* and *G*. *hirsutum* acc. TM-1 as shown in Table 1. From the phylogenetic view, one member in the diploid *G*. *raimondii* would be corresponding to one homologous gene in *G*. *arboreum* and two homologous genes (homeologs from A and D subgenome) in tetraploid *G*. *hirsutum* acc. TM-1. The inconsistency of the homologous genes among these three cotton species might result from the gene loss during their individually evolution process or assembly error in partial chromosomal regions, and need to be further confirmed. The related information of *COBLs* in *Gossypium* was summarized in Table 1.

**Table 1:** Characterization of COBL family genes identified in *G*. *raimondii* and their phylogenetic relationship in other cotton species^a^.

Gene name	Chr.^b^	Gene ID in *G*. *raimondii*	Calculated protein information			Ortholog Gene ID (Gene name) in	
			Length (a.a.)	pI	MW (kDa)	*G*. *arboreum*	*G*. *hirsutum* acc. TM-1^c^
*GrCOBL1*	D1	Gorai.002G006300	356	8.23	40.01	Cotton_A_00213 (*GaCOBL1*)	Gh_A01G0051/Gh_D01G0052 (*GhCOBL1* A_t_/D_t_)
*GrCOBL2*	D1	Gorai.002G083800	667	9.2	74.54	Cotton_A_04743 (*GaCOBL2*)	Gh_A01G0582/Gh_D01G0593 (*GhCOBL2* A_t_/D_t_)
*GrCOBL3*	D2	Gorai.005G065200	657	6.6	72.31	Cotton_A_21798 (*GaCOBL3*)	Gh_A02G0513/Gh_D02G0577 (*GhCOBL3* A_t_/D_t_)
*GrCOBL4*	D3	Gorai.003G102500	489	9.09	54.65	Cotton_A_34370 (*GaCOBL4*)	Gh_A03G0631.1/Gh_D03G0919.1 (*GhCOBL4* A_t_/D_t_)
*GrCOBL5*	D3	Gorai.003G102700	439	8.9	49.18	Cotton_A_34369 (*GaCOBL5*)	Gh_A03G0631.2/Gh_D03G0919.2 (*GhCOBL5* A_t_/D_t_)
*GrCOBL6*	D6	Gorai.010G177700	661	9.11	74.22	Cotton_A_35783 (*GaCOBL6*)	Gh_A06G1281/Gh_D06G1606 (*GhCOBL6* A_t_/D_t_)
*GrCOBL7*	D7	Gorai.001G076200	438	8.98	49.34		Gh_A07G0608/Gh_D07G0673 (*GhCOBL7* A_t_/D_t_)
*GrCOBL8*	D7	Gorai.001G208300	455	9.03	50.99	Cotton_A_33232 (*GaCOBL8*)	Gh_A07G1625/Gh_D07G1823 (*GhCOBL8* A_t_/D_t_)
*GrCOBL9*	D8	Gorai.004G063600	430	9.07	48.37	Cotton_A_03290 (*GaCOBL9*)	Gh_A08G0474 (*GhCOBL9* A_t_)
*GrCOBL10*	D10	Gorai.011G196300	624	5.43	68.66	Cotton_A_19861 (*GaCOBL10*)	Gh_A10G1503/Gh_D10G1749 (*GhCOBL10* A_t_/D_t_)
*GrCOBL11*	D11	Gorai.007G125700	656	5.21	71.69	Cotton_A_26440 (*GaCOBL11*)	Gh_A11G1019/Gh_D11G1175 (*GhCOBL11* A_t_/D_t_)
*GrCOBL12*	D11	Gorai.007G176300	450	8.59	50.28	Cotton_A_12493 (*GaCOBL12*)	Gh_A11G1468/Gh_D11G1625 (*GhCOBL12* A_t_/D_t_)
*GrCOBL13*	D11	Gorai.007G176400	433	9.05	49.23	Cotton_A_12494 (*GaCOBL13*)	Gh_A11G1469/Gh_D11G1626 (*GhCOBL13* A_t_/D_t_)
*GrCOBL14*	D12	Gorai.008G027100	446	7.77	50.33	Cotton_A_10803 (*GaCOBL14*)	
*GrCOBL15*	D12	Gorai.008G033200	442	5.42	49.76	Cotton_A_15300 (*GaCOBL15*)	Gh_D12G0298 (*GhCOBL15* D_t_)
*GrCOBL16*	D12	Gorai.008G200000	455	8.87	50.79	Cotton_A_31975 (*GaCOBL16*)	Gh_A12G1661/Gh_D12G1818 (*GhCOBL16* A_t_/D_t_)
*GrCOBL17*	D12	Gorai.008G200100	434	9.27	48.98	Cotton_A_31976 (*GaCOBL17*)	Gh_D12G1819 (*GhCOBL17* D_t_)
*GrCOBL18*	D13	Gorai.013G039400	452	7.05	50.92	Cotton_A_19405 (*GaCOBL18*)	Gh_A13G0320/Gh_D13G0359 (*GhCOBL18* A_t_/D_t_)
*GrCOBL19*	D13	Gorai.013G163300	660	8.68	73.63	Cotton_A_17907 (*GaCOBL19*)	Gh_A13G1193/Gh_D13G1487 (*GhCOBL19* A_t_/D_t_)

^a^ Genes information in *G*. *raimondii* from Paterson et al. [30], *G*. *arboreum* from Li et al. [31] and *G*. *hirsutum* from Zhang et al. [32].

^b^ Chromosome numbers D1 to D13 refer to chromosomes name of D subgenome in allotetraploid cultivated cotton species [34].

^c^ A_t_ and D_t_ were derived from the A-genome and D-genome progenitor in the tetraploid cotton.

### Chromosomal distribution and gene duplication of *COBL* genes in *G*. *raimondii*

**Fig 1:**
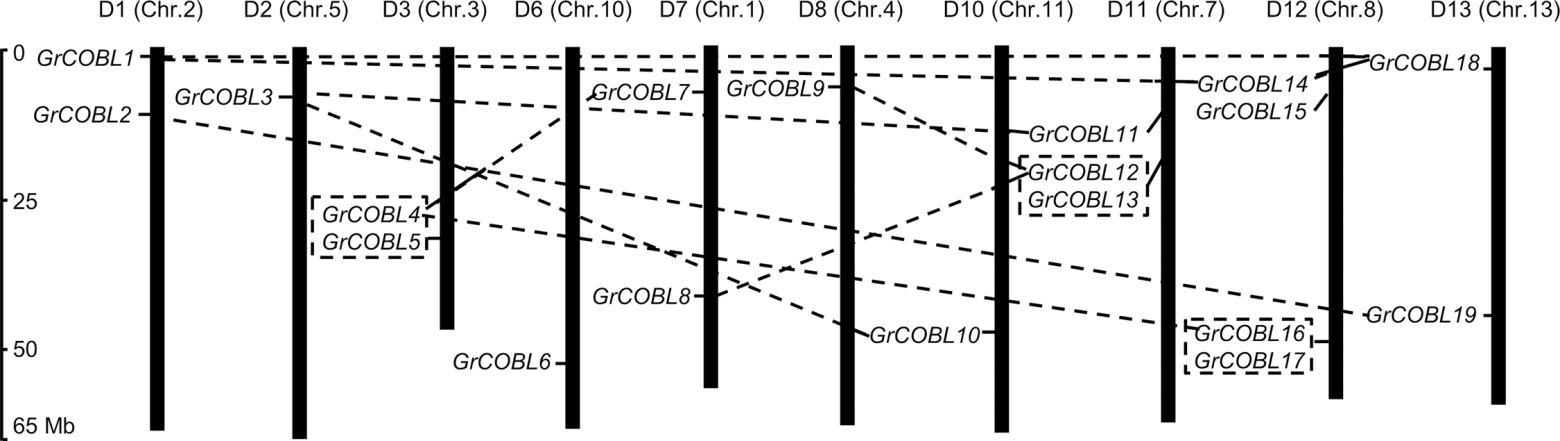
Chromosomal distribution of the *COBL* family genes in *G*. *raimondii*. The chromosome numbers were consistent with the interspecific genetic map (D1 to D13) in allotetraploid cultivated cotton species [34] and the scaffolds (Chr.1 to Chr.13) in the genomic data of *G*. *raimondii* [30]. The nomenclature of *COBLs* was based on the order of the chromosomes in *G*. *raimondii*. Lines were drawn to connect the duplicated genes.

Two sequenced diploid genomes A genome *G*. *arboreum* and D genome *G*. *raimondii* were the closest extant progenitor relatives for tetraploid cotton species. Of them, the genome sequence in *G*. *raimondii* had been well assembled and annotated [30] and the collinearity of linkage groups between *G*. *raimondii* and *G*. *hirsutum* acc. TM-1 genome was obvious [32]. So we selected preferentially *G*. *raimondii* genome information to characterize the *COBL* family genes. By integrating 13 scaffolds of the *G*. *raimondii* genome (named as Chr. 1 to Chr. 13) with the reported high-density interspecific genetic map of allotetraploid cultivated cotton species [34], we obtained the corresponding relationship of orthologs between Chr. 1 to Chr. 13 in *G*. *raimondii* and D1 to D13 chromosomes in tetraploid cotton species. As a result, 19 candidate *COBL* genes were matched to 10 scaffolds of the *G*. *raimondii* genome. Based on the order of the homologs on chromosomes, we named the *COBL* members in *G*. *raimondii* as *GrCOBL1* to *GrCOBL19*. Correspondingly, we also named *COBL* family genes *GaCOBL1* to *GaCOBL19* in *G*. *arboreum* and *GhCOBL1* to *GhCOBL19* in *G*. *hirsutum* acc. TM-1 following the orthologous relationship with the ones in *G*. *raimondii* (Table 1). The chromosomal distribution patterns of these *GrCOBL* genes were uneven. As in Fig 1, no *GrCOBL* was detected in the three chromosomes D4, D5 and D9, four *GrCOBLs* were found on the D12 chromosome, three on the D11 chromosome, two each were found on the D1, D3, D7 and D13 chromosomes, and only one each on the D2, D6, D8 and D10 chromosomes.

It was believed that whole genome duplication with genetic redundancy, chromosomal rearrangement and genome downsizing brought about a gene family a substantial expansion and evolutionary novelties [49]. The *G*. *raimondii* genome had undergone at least two rounds of genome-wide duplication [30]. To understand the mechanism of the *COBL* gene family expansion in *G*. *raimondii*, duplication events including tandem and segmental duplications were investigated by genome synteny analysis (Fig 1). Three gene pairs (*GrCOBL4*/*GrCOBL5*, *GrCOBL12*/*GrCOBL13* and *GrCOBL16*/*GrCOBL17*) were detected as tandem duplication events, with 69.5%, 64.4% and 53.5% sequence similarity respectively. In addition ten gene pairs with high similarity that were located on segmental duplicated blocks were found to be a result of segmental duplication. All these paralogous pairs had ratios of nonsynonymous to synonymous substitutions (Ka/Ks) of less than 0.2 (Table 2) implying that *COBL* family genes in *G*. *raimondii* had mainly been subject to purifying selection pressure during the evolutionary process.

**Table 2:** Nucleotide diversity and expression correlation among 10 *COBL* paralog pairs.

Paralog pairs in *G*. *raimondii* ^a^	Subgroup	Sequence similarity (%)^b^	Ka	Ks	Ka/Ks	Pearson correlation coefficient of expression pattern in *G*. *hirsutum* acc.TM-1^c^
*GrCOBL1-GrCOBL14*	Ⅰ	69.7	0.183	1.083	0.169	--
*GrCOBL1-GrCOBL18*	Ⅰ	69.9	0.176	0.997	0.177	0.000
*GrCOBL2-GrCOBL19*	Ⅱ	65.5	0.233	1.209	0.193	1.000
*GrCOBL3-GrCOBL10*	Ⅱ	83.2	0.086	0.587	0.146	0.777
*GrCOBL3-GrCOBL11*	Ⅱ	63.1	0.279	2.313	0.121	-0.030
*GrCOBL4-GrCOBL7*	Ⅰ	55.7	0.298	1.780	0.167	-0.012
*GrCOBL4-GrCOBL16*	Ⅰ	60	0.311	None	None	0.438
*GrCOBL8-GrCOBL12*	Ⅰ	84.9	0.075	0.441	0.169	-0.104
*GrCOBL9-GrCOBL12*	Ⅰ	63.5	0.263	None	None	-0.147
*GrCOBL14-GrCOBL18*	Ⅰ	77.6	0.138	0.719	0.191	--

^a^ Paralog pairs were referred to Plant Genome Duplication Database and the Ka/Ks ratio was calculated to assess the selection pressure for each paralog pair.

^b^ DNAMAN was used to calculate the sequence similarity between the paralog pairs.

^c^ Pearson correlation coefficient: r>0: positive correlation; r<0: negative correlation; r = 0: no linear correlation; --: undetectable.

### Phylogenetic and structural analysis of *COBL* genes

**Fig 2:**
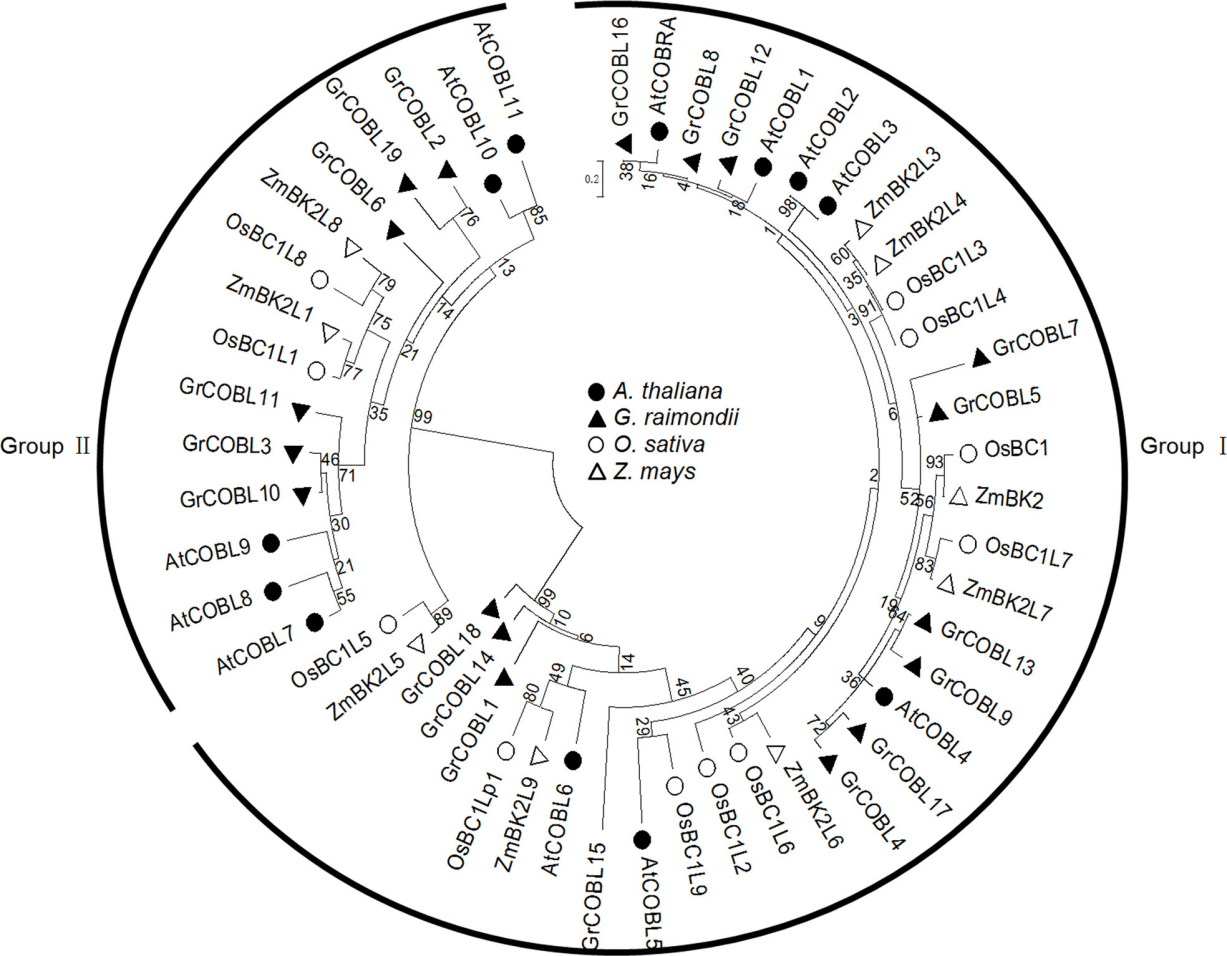
Phylogenetic analyses of *COBL* family genes in eudicot (*A*. *thaliana*, *G*. *raimondii*) and monocot (*O*. *sativa*, *Z*. *mays*). Amino acid sequences were aligned using ClustalW and the phylogenetic tree was conducted using MEGA 5.0 software with the maximum likelihood method. Four different color fonts were represented for the four species.

The overall protein sequences including *COBLs* in the eudicots (*A*. *thaliana*, *G*. *raimondii*) and monocots (*O*. *sativa*, *Z*. *mays*) were utilized to construct an unrooted tree to clarify the phylogenetic relationship of *COBL* genes from different species. Consistent with orthologs in *A*. *thaliana* and other species, the *COBL* family members were clustered into two subgroups Group Ⅰand Group Ⅱ, with phylogenetically related to *AtCOBRA* and *AtCOBL7* in *A*. *thaliana* respectively. As in Fig 2, thirteen *COBL* genes from *G*. *raimondii*, seven from *A*. *thaliana*, eight from *O*. *sativa* and six from *Z*. *mays* were clustered in Group Ⅰ and six from *G*. *raimondii*, five from *A*. *thaliana*, three from *O*. *sativa* and three from *Z*. *mays* were in Group Ⅱ. The phylogenetic analysis indicated that the *COBLs* were descendants of an ancient duplication that occurred even before the separation of monocots and dicots.

**Fig 3:**
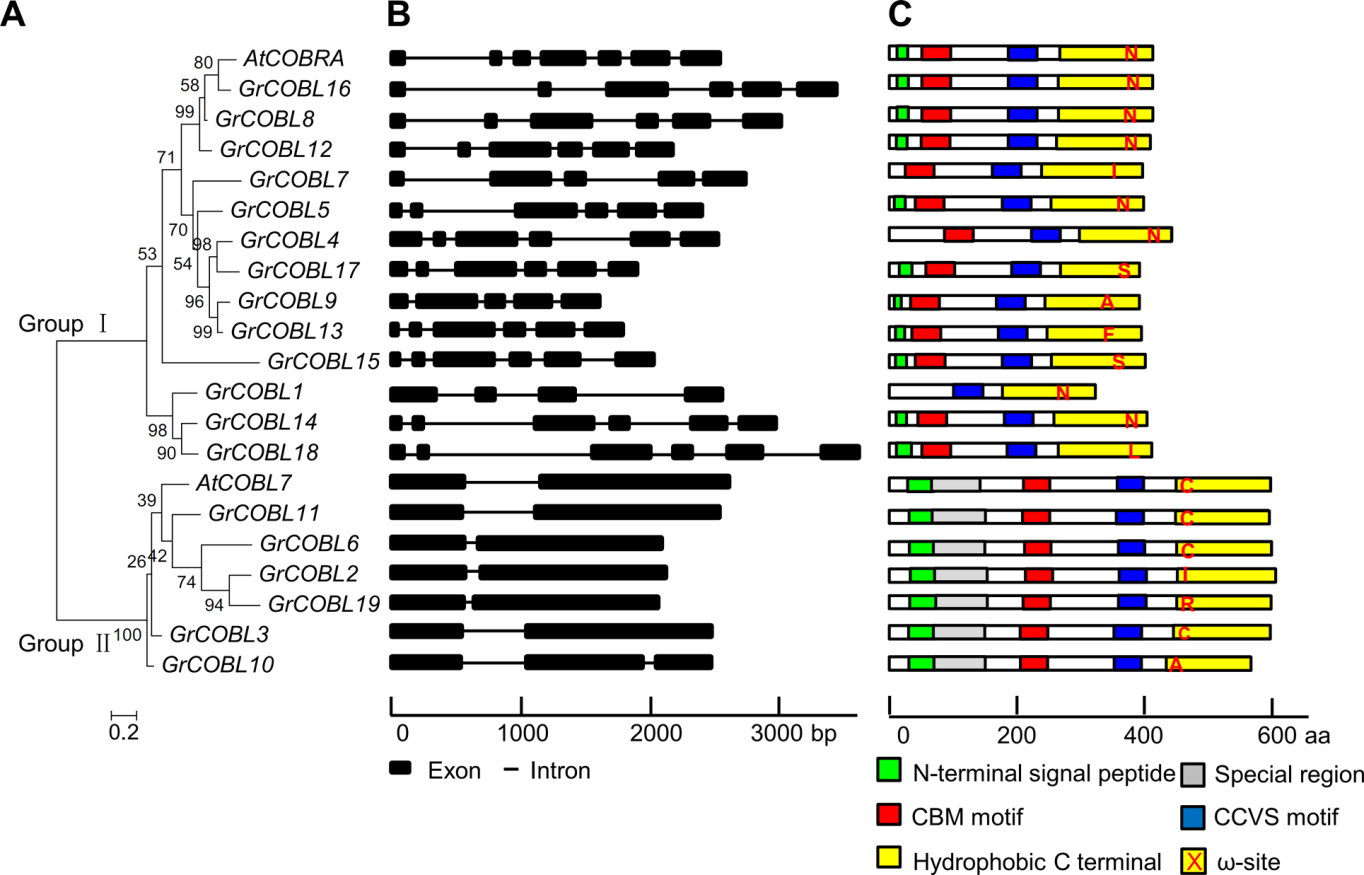
Phylogenetic relationship, gene structure and motif compositions of the *COBLs* in *G*. *raimondii* and *AtCOBRA*, *AtCOBL7* in *A*. *thaliana*. A. The phylogenetic tree was conducted using MEGA 5.0 software with the maximum likelihood method. B. Exon/intron organization of *GrCOBL* members. Black boxes and black lines represented the exons and introns. The length of nucleotides was shown below. C. Schematic representation of the conserved motifs of *GrCOBL* proteins elucidated by MEME and SMART. Each motif was marked by the different colors and the ω-sites were showed in red font. The length of amino acids was shown below.

The orthologs from three cotton species, *G*. *raimondii*, *G*. *arboreum* and *G*. *hirsutum* acc. TM-1 shared the same gene structure, thus, the *COBLs* in *G*. *raimondii* were used to give the insights into the diversity of the *COBL* family in *Gossypium* (Fig 3A and 3B). *GrCOBLs* in group Ⅰ had more introns than those in group Ⅱ. Besides, motif analysis (Fig 3C) displayed the conserved domains characterized by *COBL* gene family: the N-terminal signal peptide, the carbohydrate-binding module, a highly conserved CCVS motif and C-terminal domains that included the GPI modification motif and a hydrophobic tail, with the exception that three genes *GrCOBL1*, *GrCOBL4* and *GrCOBL7* lacked the N-terminal hydrophobic domain and *GrCOBL1* lacked the CBM. Structurally the *GrCOBLs* in these two groups displayed the divergence at the N terminus. The members in Group Ⅱ contained an extra amino acid stretch after the N-terminal signal peptide and lead to the wide diversity in the length of amino acids ranging from 356 to 667 (Table 1).

Moreover, multiple sequence alignments showed that hydrophobicity was ubiquitous for the *COBL* members, although the low sequence consistency of the N-terminal and C-terminal regions existed (S1 Fig). With the exception of the CCVS motif and the aromatic amino acids in the CBM, which were highly conserved, the majority of the residues showed characteristics that were specific to the two clustered groups. Besides, the ω-sites in GroupⅠ were close to the end of the C-terminal regions, while the ones in Group Ⅱ were close to the front of the C-terminal regions and were followed by a longer hydrophobic tail. This had not been reported previously and required to be further confirmed.

### Expression patterns of *COBL* genes in *G*. *hirsutum* acc. TM-1

**Fig 4:**
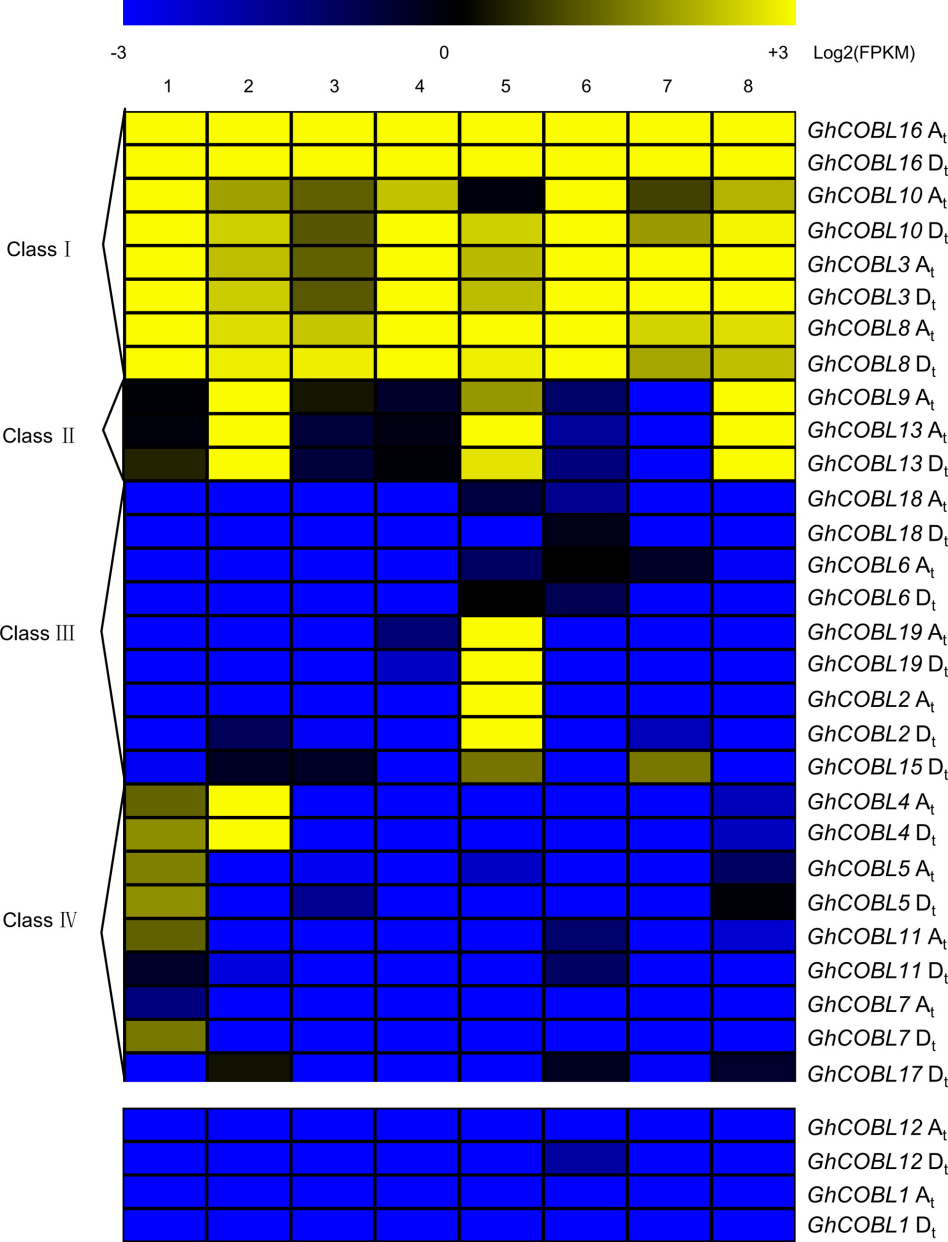
Heat map of the *COBLs* expression in different tissues of *G*. *hirsutum* acc. TM-1. The eight different tissues of *G*. *hirsutum* acc. TM-1 were involved here: 1: root; 2: stem; 3: leaf; 4: petal; 5: anther; 6: 0 DPA ovule; 7: 10 DPA fiber; 8: 20 DPA fiber. Log_2_ (FPKM) indicated the different transcriptome profiling (FPKM: fragments per kb per million mapped reads). A_t_ and D_t_ were derived from the A-genome and D-genome progenitor in the tetraploid cotton.

Based on RNA-Seq data of *G*. *hirsutum* acc. TM-1 including vegetative tissues (roots, stems and leaves), floral tissues (petals and anthers) and fibers in the development stage (0 DPA, 10 DPA and 20 DPA), we detected the expression patterns of *COBL* genes in *G*. *hirsutum* acc. TM-1. With the exception of *GhCOBL14* (not detected in *G*. *hirsutum* acc. TM-1) and *GhCOBL1*, *GhCOBL12* (FPKM<1.0 in all tested tissues), the expression patterns of other 15 *GhCOBL*s with distinguishable expression of homeologs could be grouped into four major classes (Fig 4). Four genes *GhCOBL3*, *GhCOBL8*, *GhCOBL10* and *GhCOBL16* in Class Ⅰ were highly expressed in all tissues examined with slight expression differences among the different samples. *GhCOBL9* and *GhCOBL13* in Class Ⅱ showed the predominant expression in the fiber tissue at 20 DPA. Five genes *GhCOBL2*, *GhCOBL6*, *GhCOBL15*, *GhCOBL18* and *GhCOBL19* in Class Ⅲ showed preferential expression in floral tissue with some expressed in fiber tissue. Five genes *GhCOBL4*, *GhCOBL5*, *GhCOBL7*, *GhCOBL11* and *GhCOBL17* in Class Ⅳ showed preferential expression in root or stem tissue. Generally, the similar expression pattern between homeologs in most *COBLs* were detected in different tissues in *G*. *hirsutum* acc. TM-1. Further, the gene-specific qRT-PCR was conducted to verify the expression of the 15 *GhCOBLs* in tetraploid cotton (S2 Fig) and the results of qRT-PCR were in agreement with the RNA-Seq data (S3 Fig). A correlation between the expression patterns in different tissues for the paralogous pairs was also detected (Table 2). The correlation coefficients in three *COBL* pairs (*GhCOBL2*/*GhCOBL19*, *GhCOBL3*/*GhCOBL10* and *GhCOBL4*/*GhCOBL16*) showed the positive correlation, with the expression correlation of *GhCOBL2*/*GhCOBL19* and *GhCOBL3*/*GhCOBL10* greater than 0.5. However, other pairs had no clear correlation.

**Fig 5:**
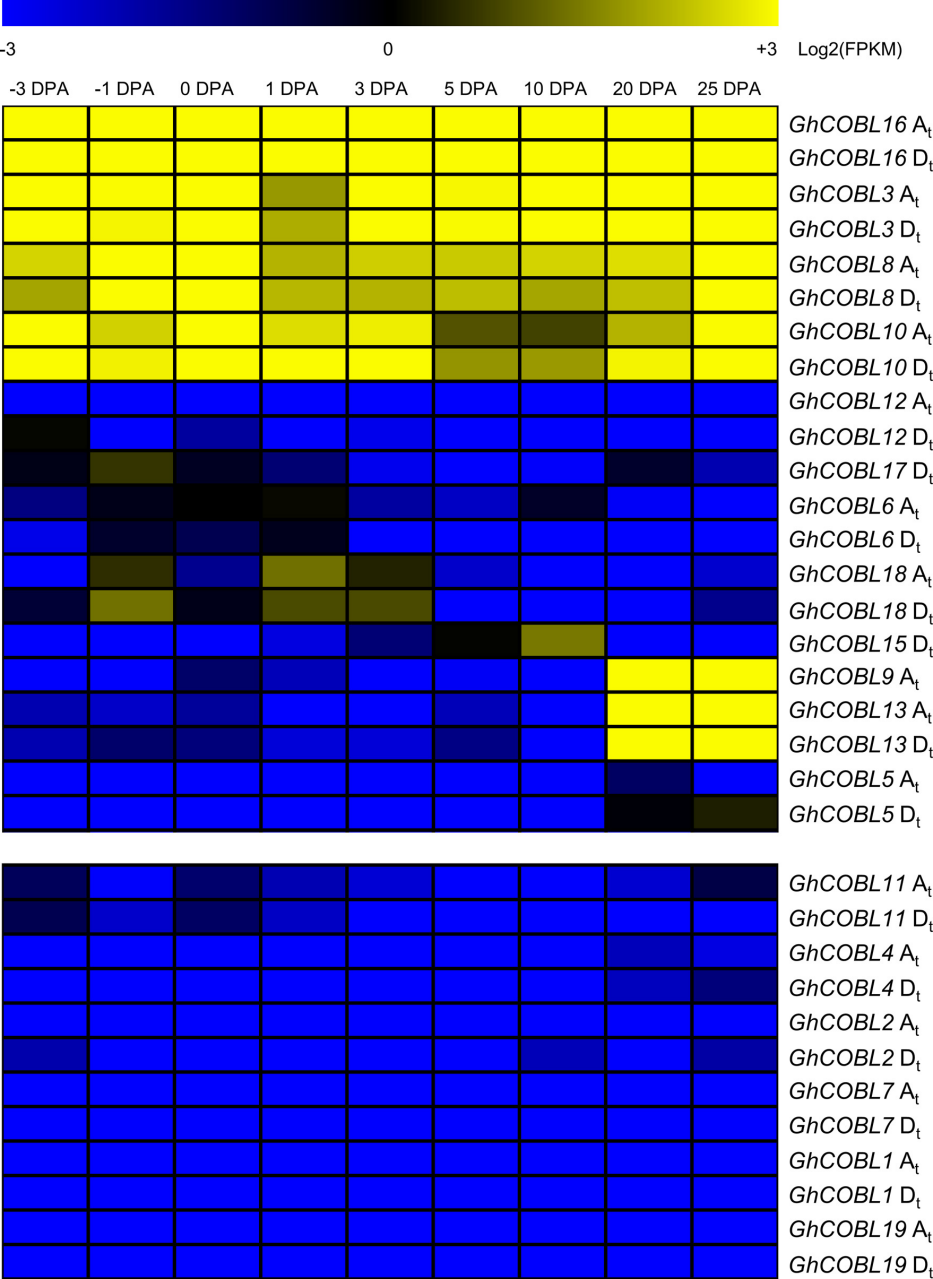
Heat map of the *COBLs* expression during the fiber developmental stages of *G*. *hirsutum* acc. TM-1. The fiber developmental stages involved in initiation (from -3 DPA to 0 DPA), elongation (from 0 DPA to 10 DPA) and secondary wall biosynthesis (from 20 DPA to 25 DPA) were sampled for detecting the expression levels of *COBL* genes during the fiber development. The relative expression levels were shown as Log_2_ (FPKM). A_t_ and D_t_ were derived from the A-genome and D-genome progenitor in the tetraploid cotton.

To elucidate the expression profiles of *COBL* genes during fiber development, we further investigated RNA-Seq data of 18 *GhCOBL* genes (with *GhCOBL14* not detected in *G*. *hirsutum* acc. TM-1) in nine samples involved in the fiber development stages of initiation (-3 DPA, -1 DPA, 0 DPA and 1 DPA), elongation (3 DPA, 5 DPA and 10 DPA) and secondary cell wall biosynthesis (20 DPA and 25 DPA) in *G*. *hirsutum* acc. TM-1 (Fig 5). With the exception of six genes *GhCOBL1*, *GhCOBL2*, *GhCOBL4*, *GhCOBL7*, *GhCOBL11* and *GhCOBL19*, which were expressed at lower levels (FPKM<1.0), the remaining genes had a varied pattern of expression during the biosynthesis of cellulose. Of these genes, four *COBLs* (*GhCOBL3*, *GhCOBL8*, *GhCOBL10* and *GhCOBL16*) were expressed at all the tested stages and maintained higher accumulation levels; Four *COBLs* (*GhCOBL12*, *GhCOBL17*, *GhCOBL6* and *GhCOBL18*) and one *COBLs* (*GhCOBL15*) showed relatively high expression levels during the initiation stage of the fiber cell and elongation stage of the fiber cell respectively than that in other tested tissues; Three *COBLs* (*GhCOBL5*, *GhCOBL9* and *GhCOBL13*) displayed higher accumulation during the secondary cell wall biosynthesis stage. The expression patterns of *GhCOBLs* in different tissues and fiber development stages displayed a dynamic change in the life cycle of fiber cells and cellulose synthesis.

### Functional prediction of two *COBL* genes and their roles in fiber quality

**Fig 6:**
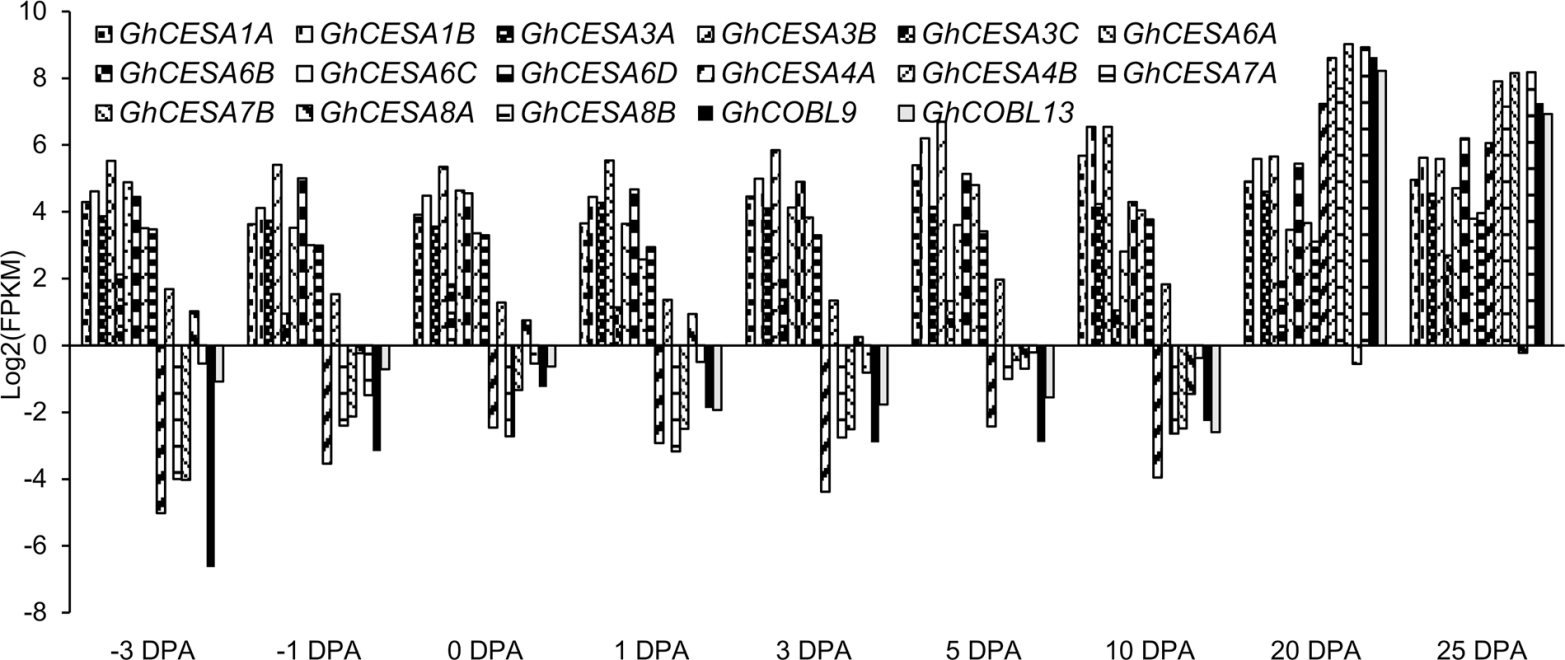
Expression patterns of *GhCOBL9* and *GhCOBL13* with the *GhCESA* family genes in *G*. *hirsutum* acc. TM-1. The X axis indicated the different fiber development stages and the Y axis indicated the expression levels.

To elucidate the relationship between *COBL* genes and fiber quality traits, we focused on the two *COBL* genes (*GhCOBL9* and *GhCOBL13*) which preferentially expressed during the secondary cell wall stage of fiber development. *Cellulose synthases* (*CESAs*) were the key factors for cellulose biosynthesis [50] and there had been at least 15 *CESA* genes which had been classified into six groups in *G*. *raimondii* [30], so we investigated the expression correlations between the two *COBLs* and *CESAs* based on the RNA-Seq data during fiber developmental stages (S3 Table and Fig 6). The results showed that *GhCOBL9*/*GhCOBL13* had nearly 100% correlation with the expression levels of *GhCESA4A*/*B*, *GhCESA7A*/*B* and *GhCESA8B*.

**Fig 7:**
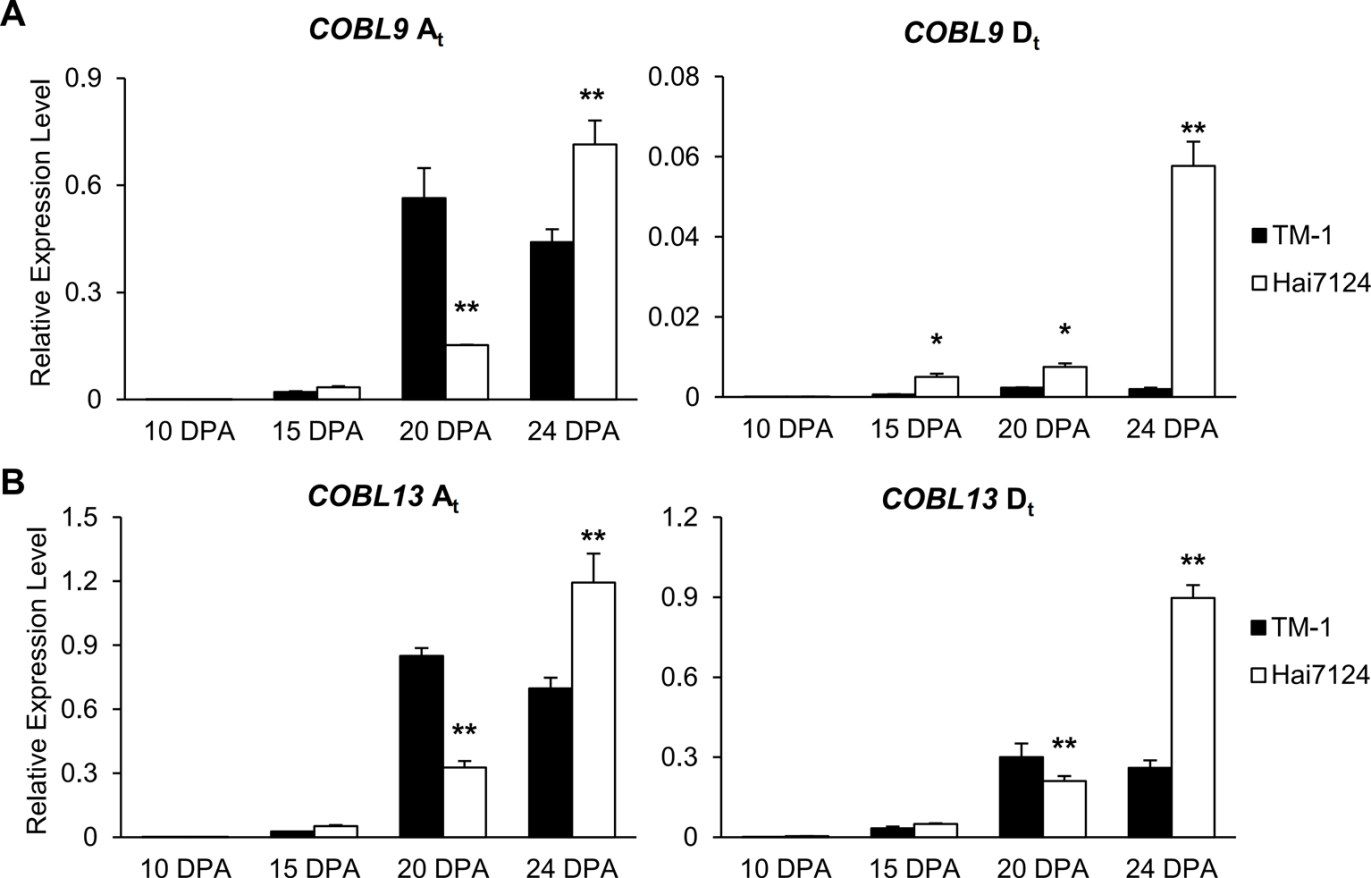
qRT-PCR analysis of *COBL9* (A) and *COBL13* (B) during the fiber development stages in TM-1 and Hai7124. The A_t_ and D_t_ were derived from A-subgenome and D-subgenome specific in tetraploid cotton species respectively. The Y axis indicated relative expression levels and the X axis indicated the different fiber development stages. “*” and “**” denoted the differences at P < 0.05 and P < 0.01 between TM-1 and Hai7124 respectively.

To further unravel the potential roles of *GhCOBL9*/*GhCOBL13* in fiber quality traits, we cloned the A and D subgenome of these two genes in *G*. *hirsutum* acc. TM-1 and their orthologs in *G*. *barbadense* cv. Hai 7124 (S4 Table). Based on the nucleotide polymorphisms of *COBL9*/*COBL13* between TM-1 D_t_ and Hai7124 D_t_, the SNP markers were developed successfully and confirmed using the SNP-PCR technology for *COBL9* D_t_ and “EcoTilling” for *COBL13* D_t_ respectively (S5 Table). For *COBL9* D_t_, the detected nucleotide polymorphism site was involved in a premature termination codon in TM-1 (TAA) but was not in Hai7124 (AAA) (S4 Fig). The SNP site also existed the similar difference within the *G*. *hirsutum* accessions (S1 Table) and the allele type “AAA” showed the favorable phenotypes related to the fiber quality traits such as FL, FM, FU (p<0.01) and FS, FE (p<0.05), especially with the longer and thinner fiber (Table 3). While for *COBL13* D_t_, the five detected SNPs were existed just between the *G*. *hirsutum* and *G*. *barbadense* but not within the *G*. *hirsutum* accessions (S1 Table; S4 Fig). Since the nucleotide polymorphism sites of *COBL9* and *COBL13* existed between TM-1 and Hai7124, we detected the expression levels of these two *COBLs* both in TM-1 and Hai7124 (Fig 7). *COBL9* A_t_ and *COBL13* A_t_ showed a high level accumulation from 10 DPA to 24 DPA both in TM-1 and Hai7124, even though the expression peak detected at 20 DPA in TM-1 but 24 DPA in Hai7124. However, compared to TM-1, *COBL9* D_t_ and *COBL13* D_t_ had an increasing accumulation from 10 DPA to 24 DPA in Hai7124 with peak at 24 DPA. In particular, *COBL9* D_t_ in Hai7124 displayed a high level accumulation, whereas the *COBL9* D_t_ in TM-1 was almost undetectable from 10 DPA to 24 DPA, which was consistent with significant correlation between *COBL9* D_t_ and fiber quality traits. As for the contribution of *COBL9*/*COBL13* from the A-subgenome to fiber qualities, we would further investigate in the future study.

**Table 3:** Correlation analysis of the allele types of *COBL9* D_t_ with fiber quality traits ^a^.

Gene Name	Fiber quality traits^b^	Anyang			Kuerle			Nanjing		
		2007	2008	2009	2007	2008	2009	2007	2008	2009
*COBL9*	FL(mm)	28.77/29.79**	29.69/30.02	27.85/28.25	29.09/29.52	29.72/30.53**	28.70/29.04	29.20/29.75*	28.95/29.10	28.38/29.07*
(TAA/AAA)	FS(cN/tex)	28.44/28.55	28.30/28.39	28.00/28.18	28.27/28.59	29.23/29.78	28.74/29.15	29.67/29.90	28.67/29.50*	28.29/28.67
	FM	4.66/4.81**	5.03/5.17*	4.41/4.61*	4.09/4.11	4.33/4.53**	4.41/4.68**	4.46/4.63*	4.13/4.30	4.84/4.89
	FE(%)	6.39/6.30	6.40/6.43	7.55/7.47	6.27/6.21	6.49/6.55	6.71/6.75*	6.26/6.24	6.33/6.36	6.63/6.66
	FU(%)	84.07/85.07**	84.51/85.01	83.61/83.92	83.55/83.84	84.47/85.11**	85.02/85.21	83.64/84.20*	83.01/83.22	84.01/84.23

^a^ Data of different fiber quality traits (the Ref./the Alt.) from natural population in *G*. *hirsutum* were analyzed with SPSS.

“*” and “**” denoted the differences at P < 0.05 and P < 0.01 between the Ref. alleles and the Alt. alleles respectively.

^b^ FL: fiber length; FS: fiber strength; FM: fiber micronaire; FE: fiber elongation; FU: fiber uniformity.

**Fig 8:**
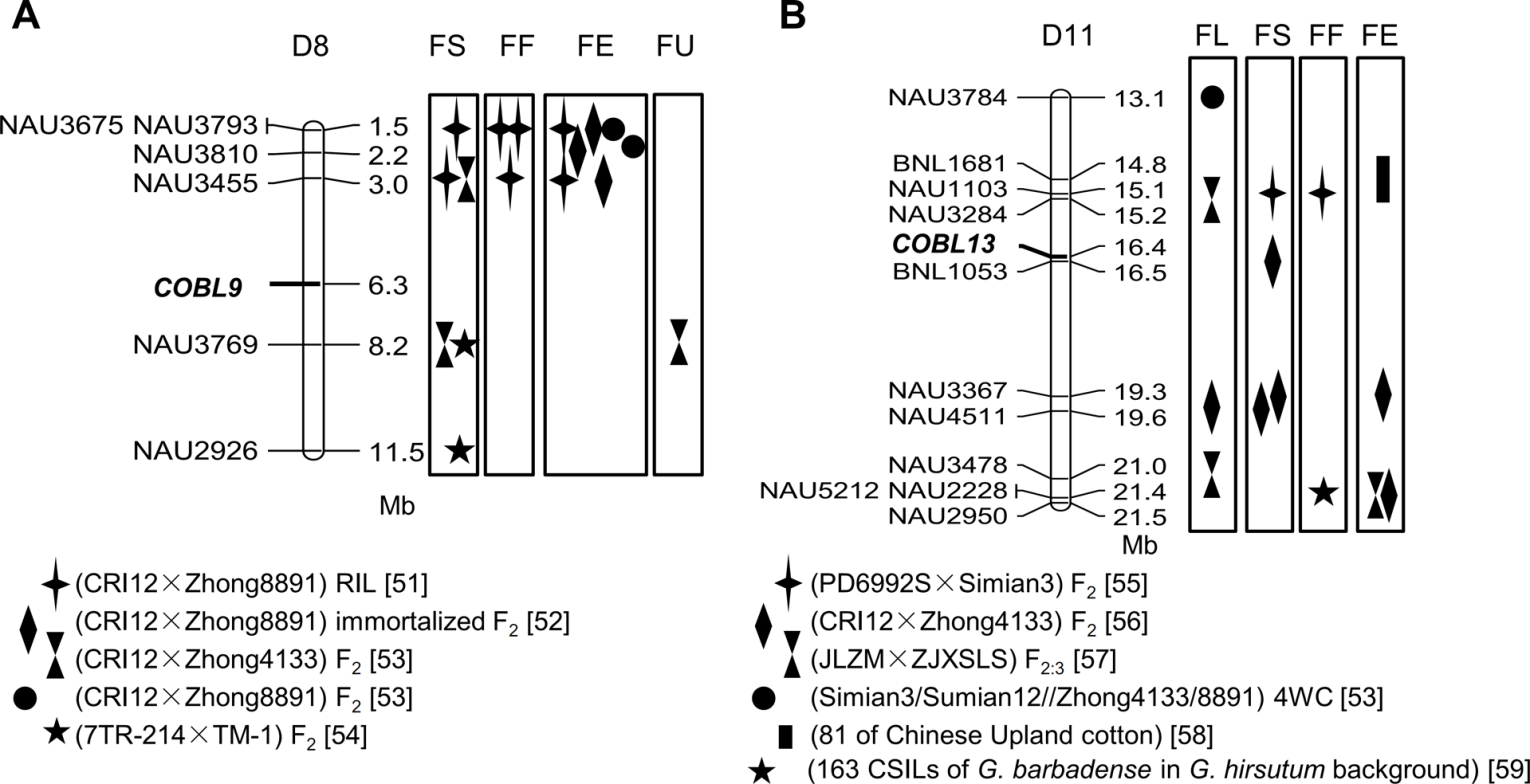
Physical integration of *GhCOBL9* and *GhCOBL13* with QTLs associated with fiber quality traits. Fiber quality traits included fiber length (FL), fiber strength (FS), fiber micronaire (FM), fiber elongation (FE) and fiber uniformity (FU). Different symbols represented the QTLs with the references followed.

QTLs related to fiber quality traits were also employed to predict the roles of *GhCOBL9* and *GhCOBL13*. By aligning the genome scaffolds in *G*. *hirsutum* acc. TM-1, *GhCOBL9* D_t_ and *GhCOBL13* D_t_ were mapped on the D8 and D11 chromosomes. Therefore, we further searched the QTLs flanking the target sites with ± 5 Mb intervals. As in Fig 8, both *GhCOBL9* D_t_ and *GhCOBL13* D_t_ were co-localized with the QTLs involved in several cotton fiber quality traits [51–59].

## Discussion

### *COBL* gene family was highly conserved in different species

The *COBRA-Like* gene family shared the CBM motif, CCVS motif, an N-terminal signal peptide and highly hydrophobic C terminus and had been identified in four species. These *COBL* genes could be classified into two groups GroupⅠand Group Ⅱ. In this study we systematically identified *COBL* members in three sequenced cotton species: 19 in *G*. *raimondii*, 18 in *G*. *arboreum* and 33 in *G*. *hirsutum* acc. TM-1. Consistent with other species, *COBL* family members in *Gossypium* were also classified into two groups, with Group Ⅱ carrying an amino acid stretch of unknown function after the N terminus and a longer hydrophobic tail following the ω-site.

Phylogenetic analysis showed that group Ⅰ and group Ⅱ contained the *COBLs* both from monocots and dicots (Fig 2), indicating that the *COBL* family members were descendants of an ancient duplication that occurred even before the separation of monocots and dicots. However, numerous duplications occurred after the divergence of monocots and dicots with more members of the *COBL* family in dicots than in monocots. In general, there was high sequence consistency of *COBLs* between *G*. *raimondii* and *A*. *thaliana*, however some exceptions detected (Fig 2). For example, *GrCOBL5* and *GrCOBL7* had relatively distant evolutionary relationships with the *A*. *thaliana* branch in Group Ⅰ, which might result from the additional duplication event that occurred in *Gossypium*, although *A*. *thaliana* and *Gossypium* both underwent the two common duplication events (β, α) [60–61]. Gene duplication in *Gossypium* might have played a crucial role in driving evolutionary novelty and increasing adaptation to new environments by functional diversification.

### *COBL* gene family showed highly functional diversity in different tissues of *Gossypium*

In the study, *COBL* genes in *G*. *hirsutum* acc. TM-1 were found to have diverse expression patterns in vegetative tissues, floral tissues and developing fibers. As an example, *GhCOBL2* and *GhCOBL19* exhibited a unique and high expression pattern in the anther; while *GhCOBL4* showed preferential expression in the stem (Fig 4). These results implied *COBL* genes played diverse functions in different cotton tissues. Actually, the diverse roles of *COBLs* had been elucidated in other plants. For instance, in *A*. *thaliana*, the functions of *COBL* genes were focused on two aspects. One represented by *AtCOBRA* was considered to have a significant impact on the orientation of cell expansion [62] and the other represented by *AtCOBL4* was largely responsible for cellulose crystallinity status and secondary cell wall reconstruction [19]. In addition, it had been suggested that *OsBC1L* genes, the orthologs of *AtCOBL4*, performed a range of functions and participated in a various developmental processes in rice [29]. The functional divergence of *COBLs* could also be observed in the different developmental stages of fiber formation. For example, *GhCOBL15* exhibited high transcript level in 10 DPA fibers but was low in 20 and 25 DPA fibers. Conversely, *GhCOBL9* and *GhCOBL13* were highly expressed in 20 and 25 DPA fibers but were low in 10 DPA fibers (Fig 6). These results indicated *GhCOBL15* and *GhCOBL9*/*GhCOBL13* might have the different functions in fiber developmental process. The different expression pattern of *COBL* genes could be indicative of their diverse functions in different tissues or developmental processes.

Interestingly, the functions of *COBLs* might be different from their phylogenetic clusters. We observed that the *GhCOBLs* with the differential expression patterns might come from the same phylogenetic group and the *GhCOBLs* with the similar expression patterns also might belong to the different phylogenetic groups (Figs 4 and 5). For example, four *GhCOBL* genes, *GhCOBL8* and *GhCOBL16* in group Ⅰ, and *GhCOBL3* and *GhCOBL10* in groupⅡ, showed similar expression patterns. Besides, *AtCOBRA* was expressed in most tissues in *A*. *thaliana*, while *cobra* mutant plants only had an altered phenotype in the root. *GrCOBL8* and *GrCOBL16* were clustered in the same branch as *AtCOBRA* and were preferentially expressed in a variety of tissues. Their functional mechanisms in cotton remained to be verified.

### *GhCOBL9* and *GhCOBL13* potentially affected the cotton fiber quality

Cotton was an important raw material for the textile industry worldwide. During the development of cotton fiber, the deposition of cellulose determined the thickness of cell wall, which was the structural basis for fiber strength. Based on the reasons below, we could conclude that *GhCOBL9* and *GhCOBL13* play important roles during the stages of fiber formation.

Firstly, *GhCOBL9* and *GhCOBL13* were much higher expressed in fibers during the stages of secondary cell wall biosynthesis (Figs 4 and 5) and had a significantly high coexpression pattern with *GhCESA4*, *GhCESA7* and *GhCESA8* (Fig 6), which were involved in cellulose formation of secondary cell wall [63]. The functional roles of both *GhCOBL9* and *GhCOBL13* orthologs in other species had been revealed previously. *irx6* (a T-DNA mutant of *AtCOBL4*) in *A*. *thaliana* exhibited abnormal secondary wall thickening and reduced stem strength [26] similar to the effect of *BC1* in *O*. *sativa* [27] and *BK2* in *Z*. *mays* [28]. It was reasonable that *GhCOBL9* and *GhCOBL13* which showed high amino acid sequence similarities to *AtCOBL4* might play the similar role in cotton fiber secondary cell wall thickening.

Secondly, correlation analysis revealed that nucleotide polymorphism of *GhCOBL9* D_t_, similar to Hai7124 lineage, had significantly positive correlations with several fiber quality traits, seemed bring the favorable fiber quality in *G*. *hirsutum* (Table 3). Although no nucleotide polymorphism sites were detected for *GhCOBL13* within *G*. *hirsutum* accessions, both of these two *COBLs* had higher expression levels in Hai7124 which showed the superior fiber quality than in TM-1 (Fig 7). Furthermore, it was demonstrated that *GhCOBL9* and *GhCOBL13* were co-located with the QTLs related to fiber quality reported previously (Fig 8). These findings collectively indicated *GhCOBL9* and *GhCOBL13* exerted an important role in fiber quality. Since modern agricultural mechanization requires higher fiber qualities in cotton, it is reasonable to make full use of *GhCOBL9* and *GhCOBL13* for improving the fiber quality and enhancing the durability of textiles by transgenic technology or molecular marker-assisted breeding.

## Supporting Information

S1 Fig Multiple sequence alignments of 19 *COBL* members in *G*. *raimondii* and *AtCOBRA*, *AtCOBL7* in *A*. *thaliana*. Multiple sequence alignments were carried out with ClustalX 1.83. The conserved motifs were marked by the boxes with different colors and the ω-sites were showed in red font. “*” denoted the aromatic amino acids in the CBM region.
(TIF)

S2 Fig Expression patterns of *COBL* members based on qRT-PCR analysis of *G*. *hirsutum* acc. TM-1. The X axis indicated the different tissues and organs of *G*. *hirsutum* acc. TM-1 and the Y axis indicated relative expression levels of *GhCOBL* members. The cotton histone3 (AF024716) gene was used as the reference gene and the error bars were calculated based on three biological replicates using standard deviation (SD). 1: root; 2: stem; 3: leave; 4: petal; 5: anther; 6: 0DPA ovule; 7: 10DPA fiber; 8: 20DPA fiber.
(TIF)

S3 Fig Correlation analysis of expression pattern between the FPKM of expression profiling and relative expression level of qRT-PCR for *GhCOBL* members. The X axis indicated the FPKM of expression profiling and the Y axis indicated relative expression level of qRT-PCR.
(TIF)

S4 Fig The different PCR fragments and sequencing analysis of the polymorphic sites between the two different allele types for *COBL9* and *COBL13* respectively. A and B: Distinct fragments of *GhCOBL9* and *GhCOBL13* in the two different allele types revealed by denaturing gel electrophoresis via SNP-PCR (A) and EcoTilling analysis (B). “1–12” stand for the randomly selected individuals in *G*. *hirsutum* (including 1: 70-29-5, 2: Bao6716, 3: BaZhou5628, 4: ChangRong67-12, 5: Coker139, 6: GP137, 7: Ji91-12, 8: Zhong4612YaH, 9; HuBeiSongZiDaLing, 10: Zhong507145, 11: ZhongZhiBD89 and 12: ZhongZi10Hao) and “13–16” stand for the four lines in *G*. *barbadense* (including 13:E24-33891, 14: E24-33892, 15: Hai7124 and 16: Yinzi6022). “*” denoted that each DNA sample was mixed and hybridized with TM-1 (in 1: 1 ratio). C and D: Polymorphic sites of *GhCOBL9* (C) and *GhCOBL13* (D) were subsequently confirmed by sequencing and nucleotide polymorphisms were marked in orange shadows in each site.
(TIF)

S1 Table Information on 289 accessions used for correlation analysis of the targeted genes with fiber quality traits. (XLS)

S2 Table Primers information for PCR amplification, qRT-PCR and EcoTilling analysis of *COBL* members. The R' primer of EcoTilling was to conduct the second PCR amplification for amplifing the density and specificity.
(XLS)

S3 Table Pearson correlation coefficients of expression pattern between *GhCOBLs* and *GhCESAs* in different fiber developmental stages. The pearson correlation coefficient: r>0: positive correlation; r<0: negative correlation; r = 0: no linear correlation. The genes in yellow shadow have the lowest expression level in all tested tissues in fiber developmental stages.
(XLS)

S4 Table Sequence information on *COBL9* and *COBL13* in cotton. (XLS)

S5 Table Nucleotide polymorphism of *COBL9* and *COBL13* in cotton. (XLS)
