# Supplementary figures and images for: Comprehensive Analysis of the COBRA-Like (COBL) Gene Family in Gossypium Identifies Two COBLs Potentially Associated with Fiber Quality
Source: PLoS One. 2015 Dec 28;10(12):e0145725. doi: 10.1371/journal.pone.0145725 (PMC4692504; doi:10.1371/journal.pone.0145725)

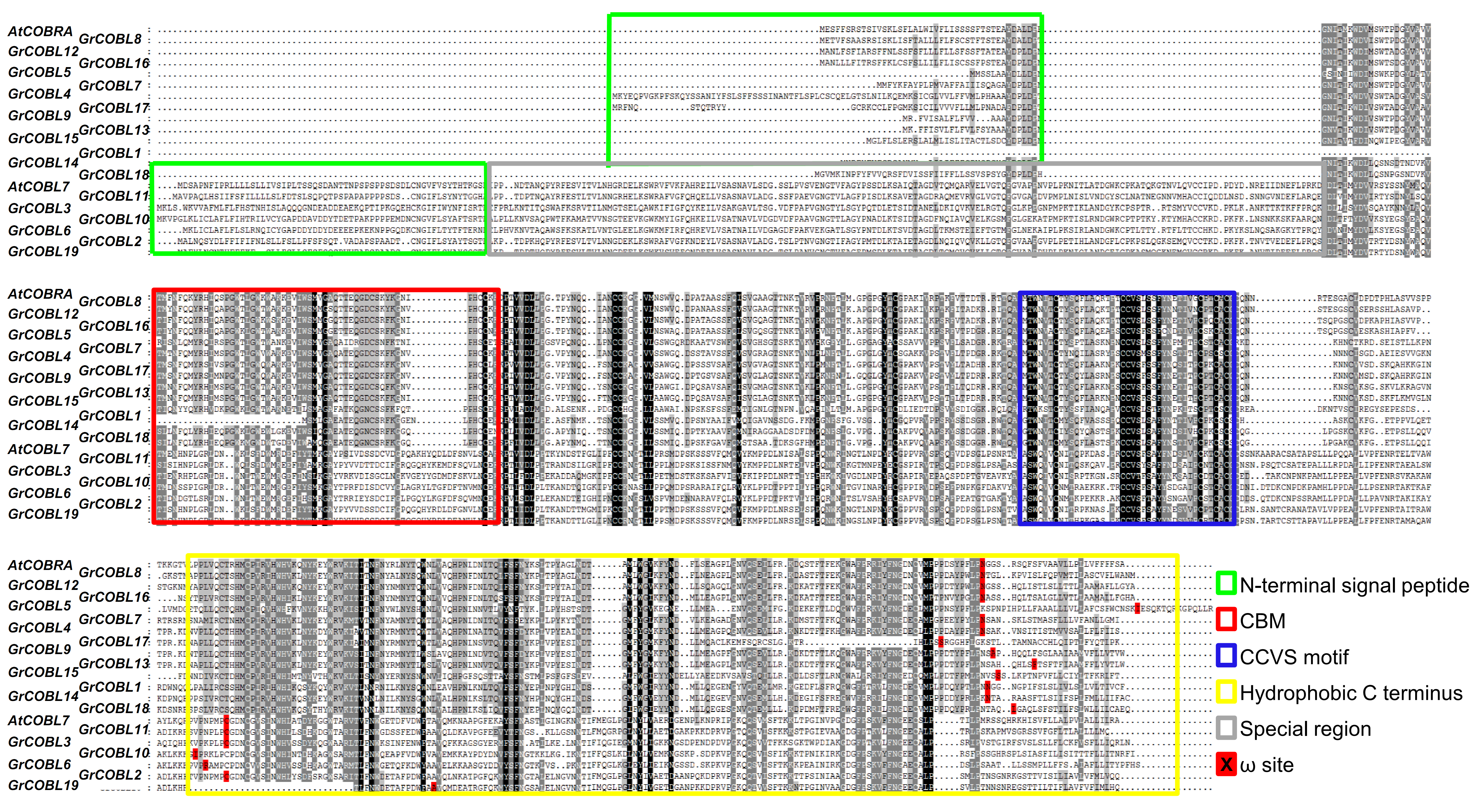

Supplement: S1 Fig — Multiple sequence alignments were carried out with ClustalX 1.83. The conserved motifs were marked by the boxes with different colors and the ω-sites were showed in red font. “*” denoted the aromatic amino acids in the CBM region. (TIF) [file pone.0145725.s001.tif]

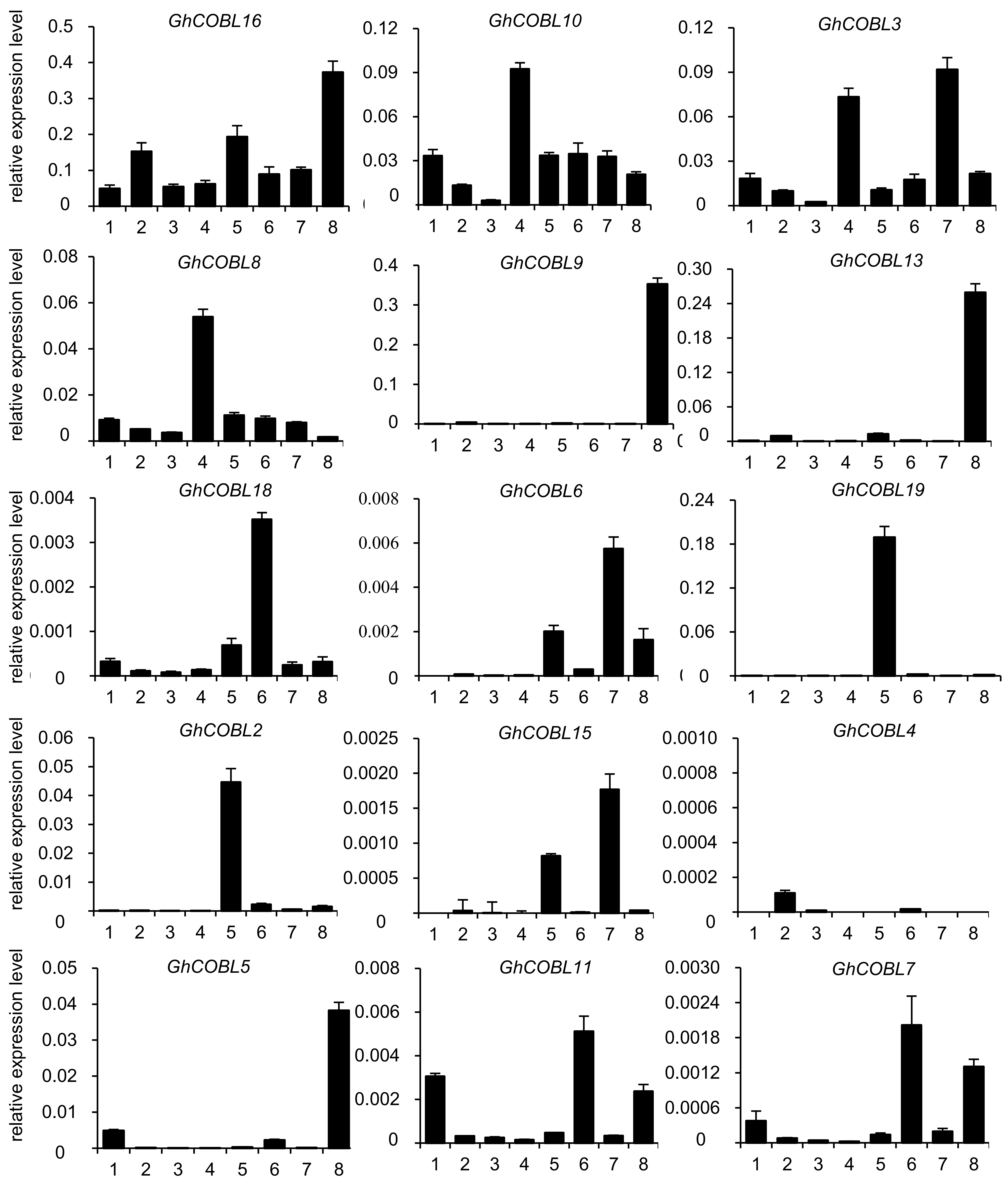

Supplement: S2 Fig — The X axis indicated the different tissues and organs of G. hirsutum acc. TM-1 and the Y axis indicated relative expression levels of GhCOBL members. The cotton histone3 (AF024716) gene was used as the reference gene and the error bars were calculated based on three biological replicates using standard deviation (SD). 1: root; 2: stem; 3: leave; 4: petal; 5: anther; 6: 0DPA ovule; 7: 10DPA fiber; 8: 20DPA fiber. (TIF) [file pone.0145725.s002.tif]

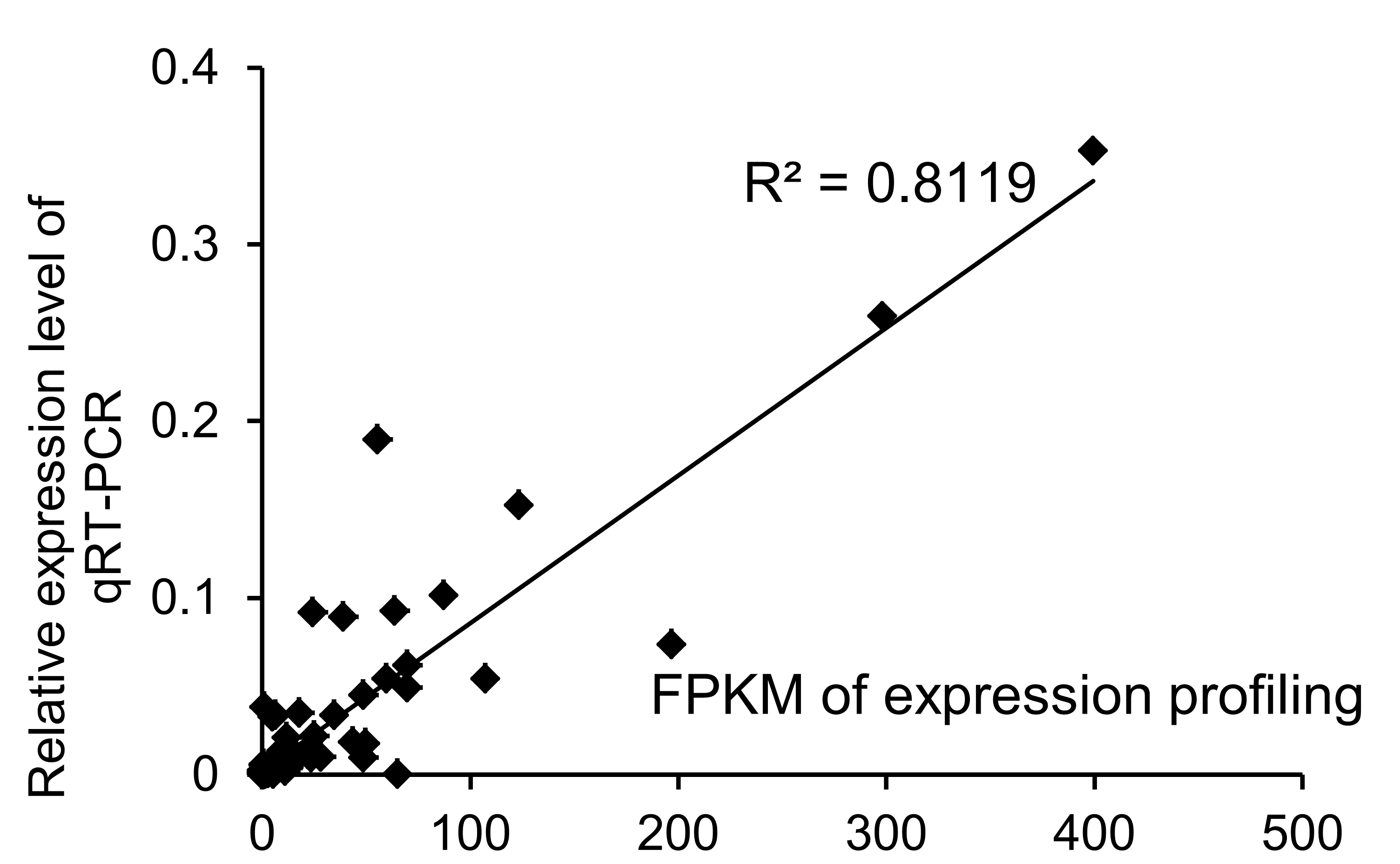

Supplement: S3 Fig — The X axis indicated the FPKM of expression profiling and the Y axis indicated relative expression level of qRT-PCR. (TIF) [file pone.0145725.s003.tif]

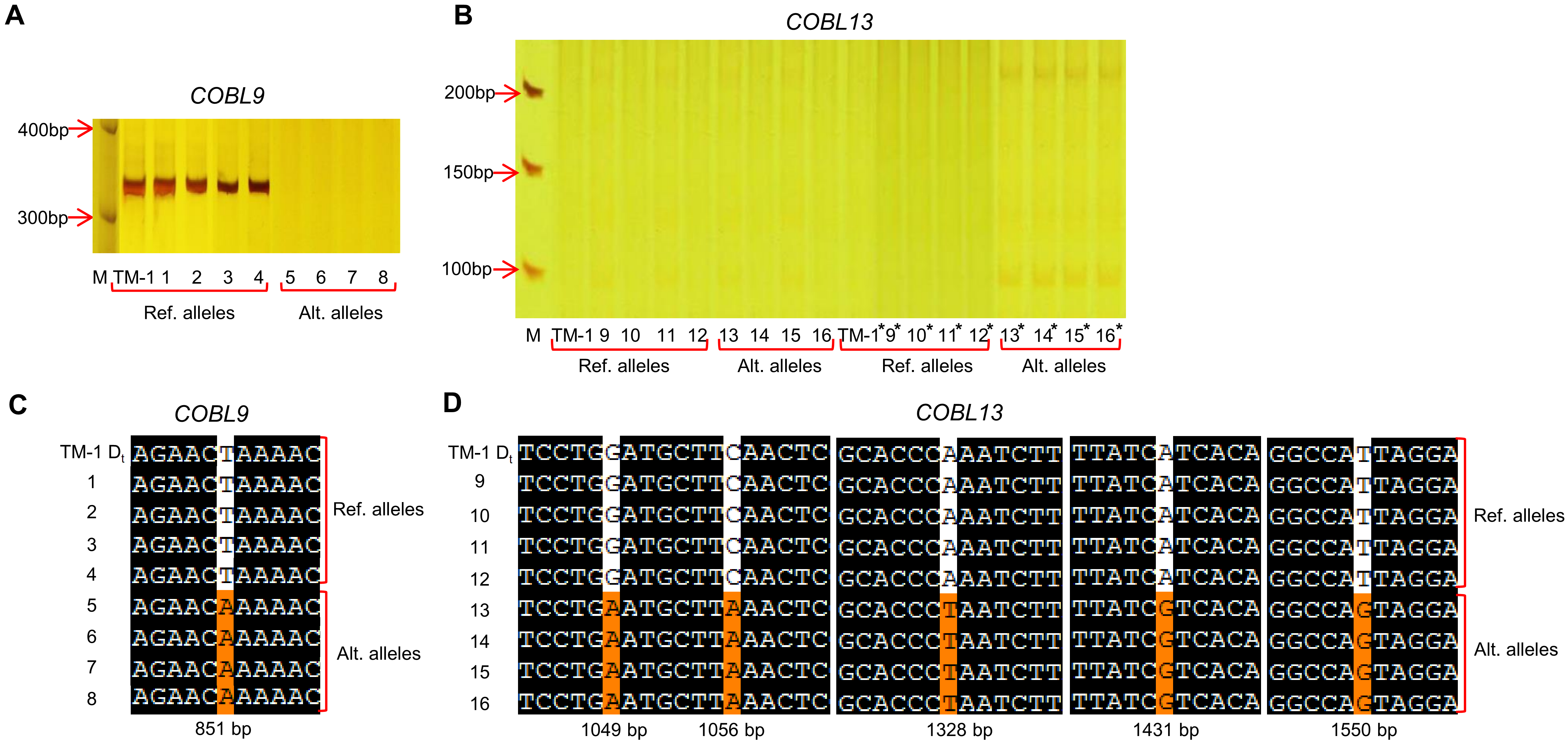

Supplement: S4 Fig — A and B: Distinct fragments of GhCOBL9 and GhCOBL13 in the two different allele types revealed by denaturing gel electrophoresis via SNP-PCR (A) and EcoTilling analysis (B). “1–12” stand for the randomly selected individuals in G. hirsutum (including 1: 70-29-5, 2: Bao6716, 3: BaZhou5628, 4: ChangRong67-12, 5: Coker139, 6: GP137, 7: Ji91-12, 8: Zhong4612YaH, 9; HuBeiSongZiDaLing, 10: Zhong507145, 11: ZhongZhiBD89 and 12: ZhongZi10Hao) and “13–16” stand for the four lines in G. barbadense (including 13:E24-33891, 14: E24-33892, 15: Hai7124 and 16: Yinzi6022). “*” denoted that each DNA sample was mixed and hybridized with TM-1 (in 1: 1 ratio). C and D: Polymorphic sites of GhCOBL9 (C) and GhCOBL13 (D) were subsequently confirmed by sequencing and nucleotide polymorphisms were marked in orange shadows in each site. (TIF) [file pone.0145725.s004.tif]
